# Supplementary material for: Heterogeneous encoding of temporal stimuli in the cerebellar cortex
Source: Nat Commun. 2023 Nov 21;14:7581. doi: 10.1038/s41467-023-43139-9 (PMC10663630; doi:10.1038/s41467-023-43139-9)
Supplement: Supplementary file 1 — Supplementary Information [file 41467_2023_43139_MOESM1_ESM.pdf]

Supplementary figures:

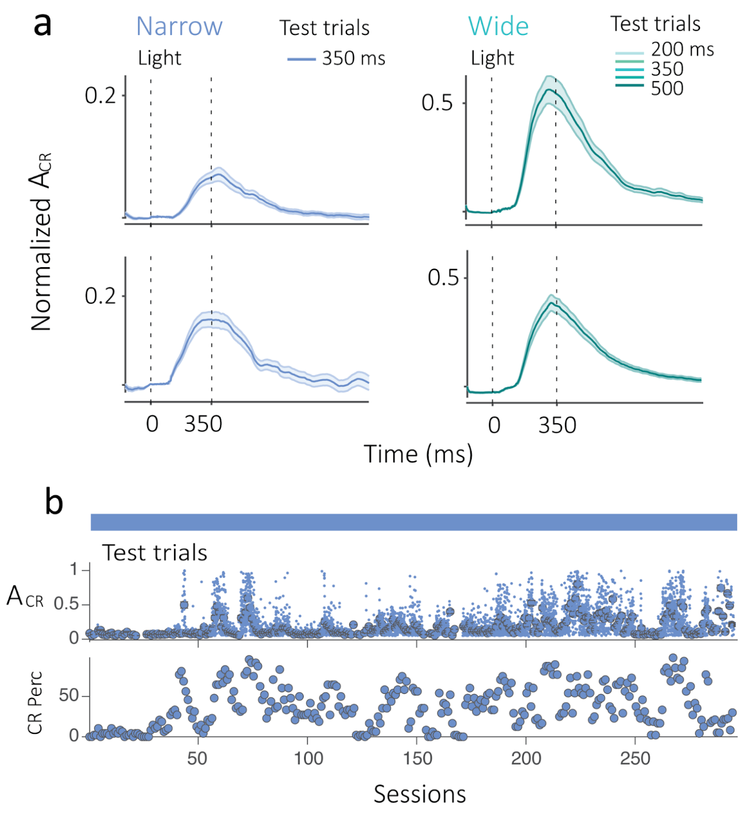

<u>Supplementary Figure 1: Behavioral training and predictive eyelid responses</u>. a) Average normalized eyelid trace examples for individuals in the Narrow (blue) or Wide condition (green) on test trials only. Error bars represent standard error. Time of light cue is indicated by a dashed line (0 ms). The average interval of anticipated airpuff(s) for both conditions (350 ms) is also indicated. b) Training regime of an individual mouse. Top: An example of the development of the conditioned response amplitude on test trials over sessions. Below: Example of development of CR percentage across sessions. Error bars represent standard error.

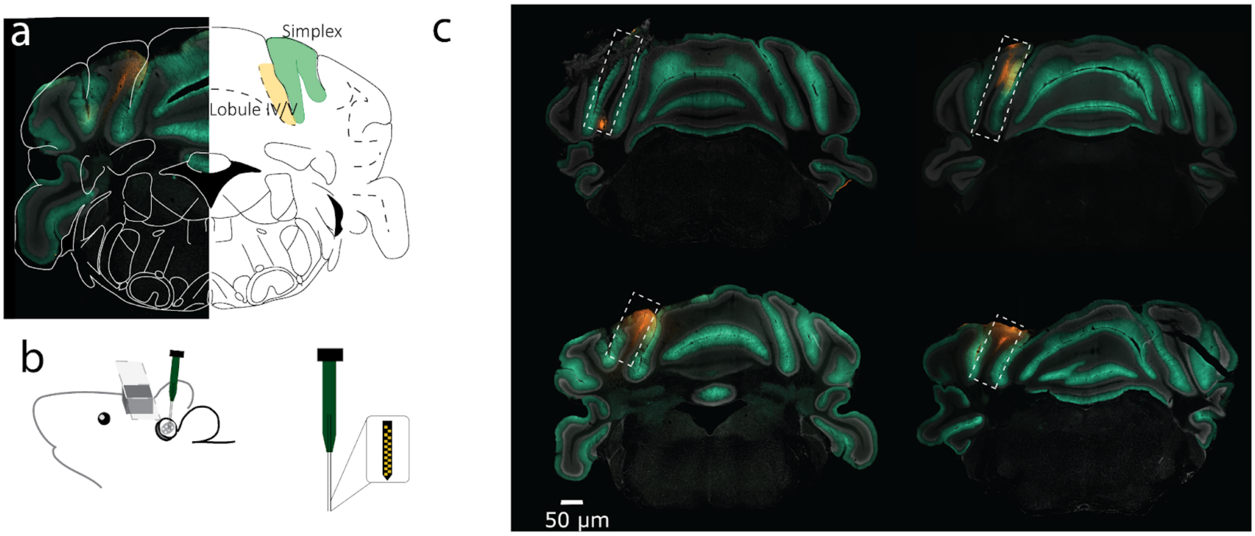

<u>Supplementary Figure 2: Location of electrophysiological recordings.</u> a) Summary of probe entry locations as marked with DiI at grid point epicenter using a single shank on the last day of recording. The entry point consistently lies between Lobule V and VI. b-c) Recordings were made from ventral (max depth -2200 um) to dorsal locations (max depth -300 um) with nonlinear double-shank electrodes, here shown in four mice. Note that perfusion was not performed on the same day after track-marking since animals continued to train on the Wide condition for several days for behavioral analysis.

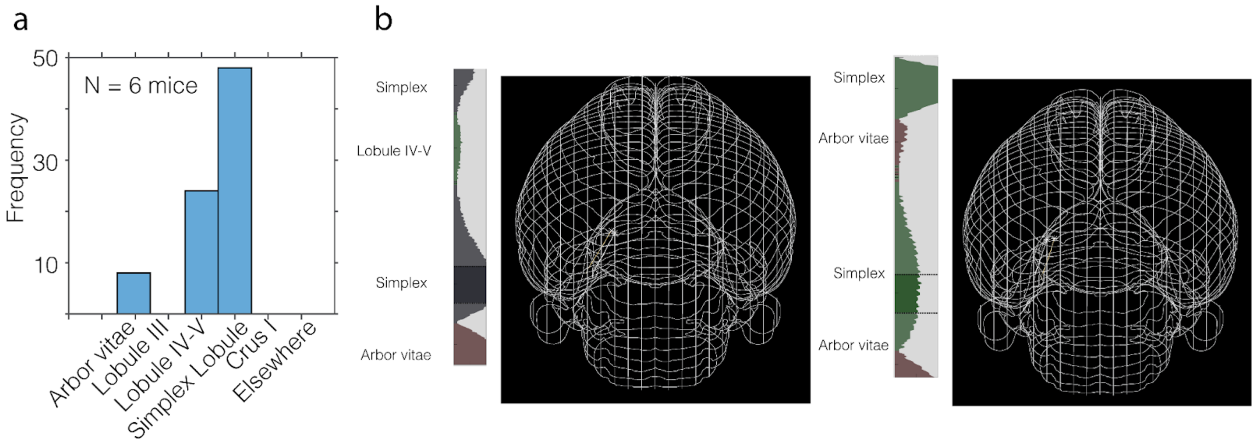

Supplementary Figure 3: Anatomical locations of probe. a) Histogram of locations of detected probe track points aligned to Allen Common coordinate framework (CCF). b) Allen CCF probe track analysis examples and probe tracks marked in a 3D mesh after registration with the Allen CCF framework for two mice.

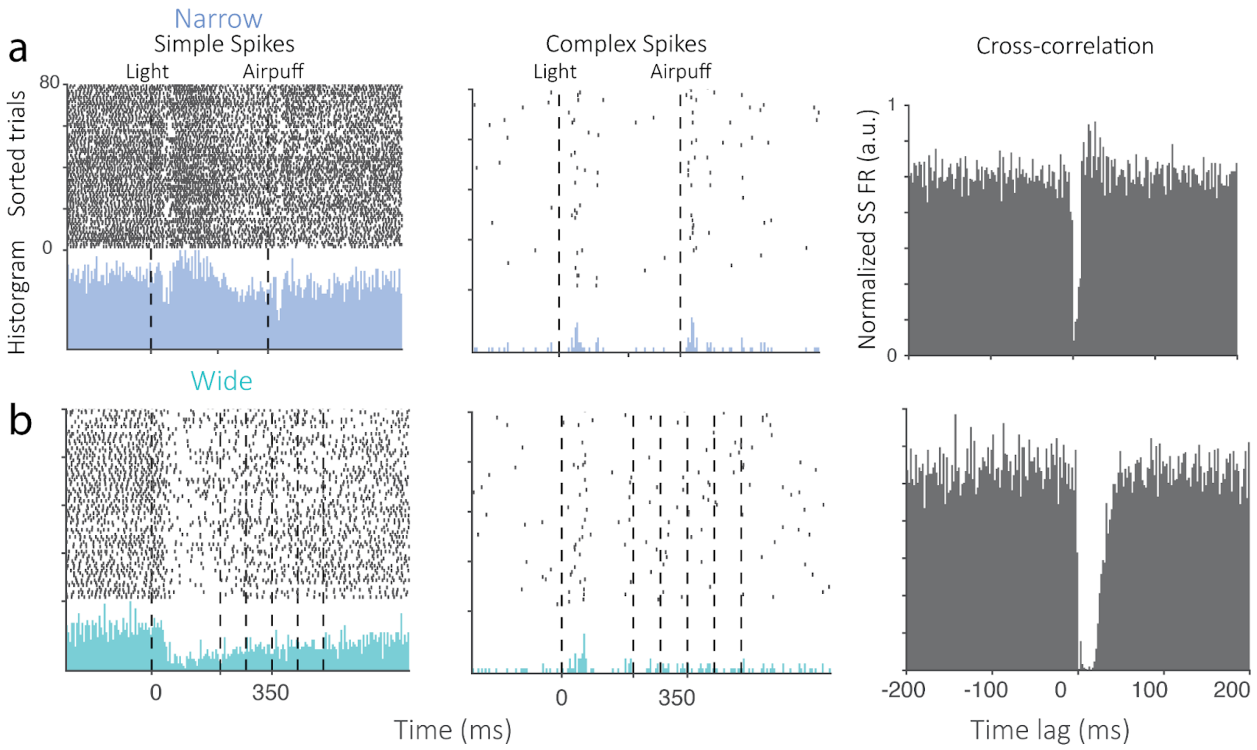

<u>Supplementary figure 4: Physiological identification of Purkinje cells.</u> a) Examples of rasters obtained during the Narrow condition (blue) from Purkinje cell simple spikes (left), complex spikes (middle), and cross-correlograms (right) that reveal a 10-20 ms suppression of simple spikes by complex spikes. b) same as a) but for the Wide distribution condition.

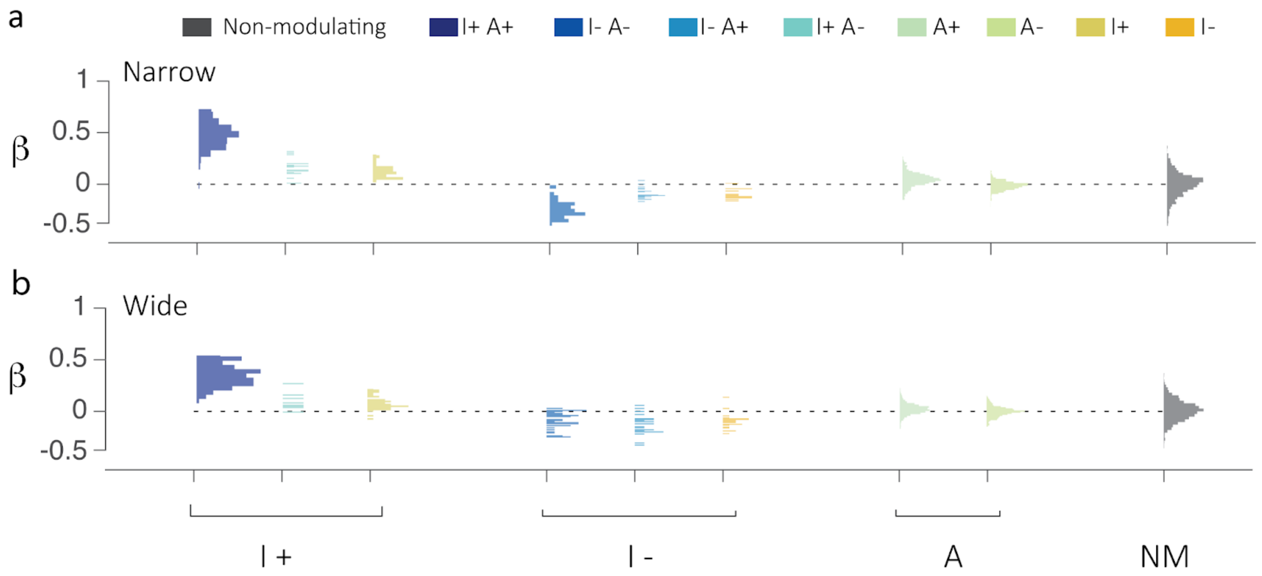

<u>Supplementary figure 5: Classification of neurons based on interval-related and airpuff-related responses.</u> Histograms of the epoch-related GLM weights (β) for each class for the a) Narrow and b) Wide conditions for all mice (N = 6).

Example motifs for cerebellar cortical heterogeneity

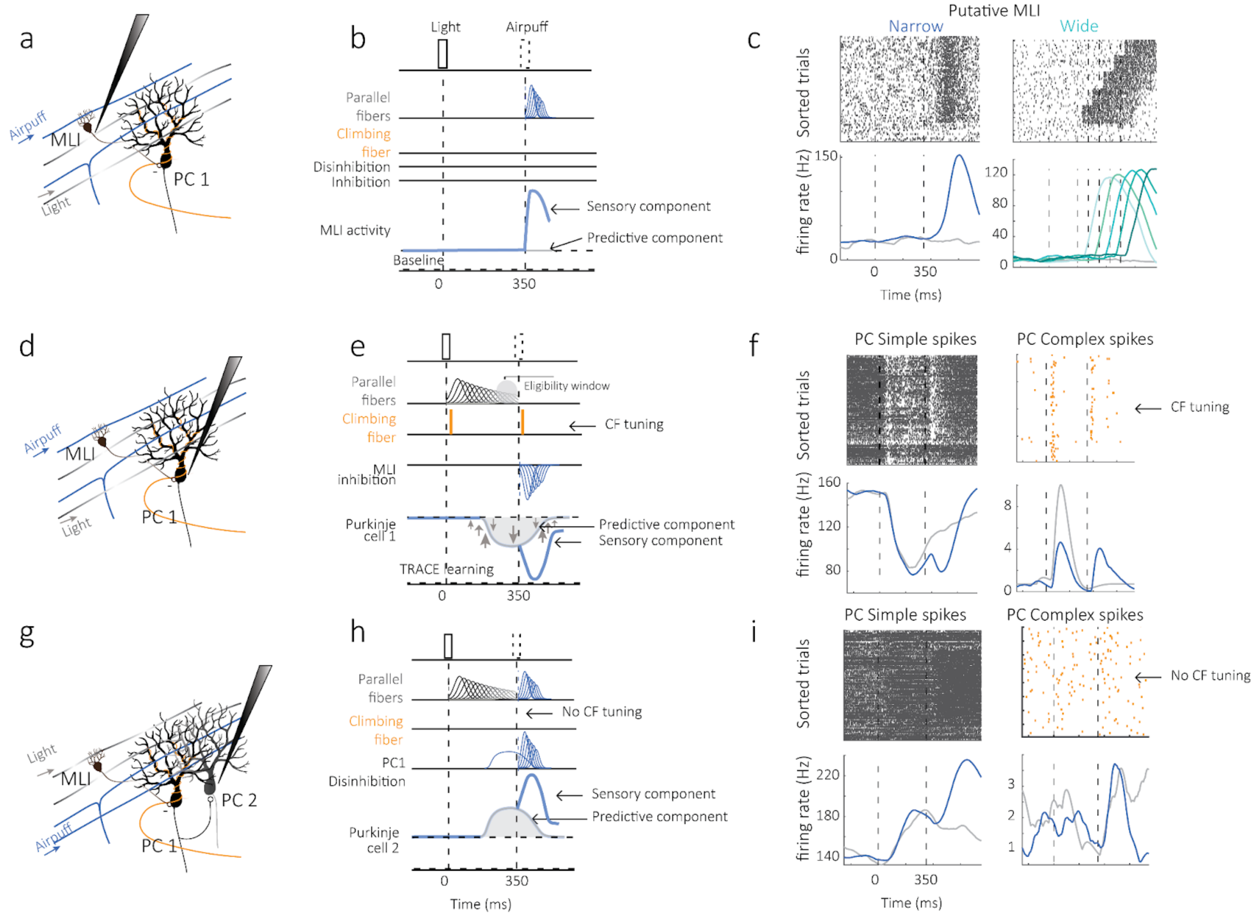

<u>Supplementary figure 6: Motif illustrations.</u> a) Model illustrations for the activity of a molecular layer interneuron (MLI) that receives airpuff sensory inputs through parallel fibers (PF). b) Such an MLI would only respond to the sensory input from the airpuff above its own baseline activity. c) Putative MLIs recorded during Narrow and Wide conditions that match this description. d,e) According to the TRACENet model, Purkinje cells (PC) that receive an airpuff-related climbing fiber (CF) signal are likely to show suppression in their activity during the range of the temporal distribution. In this example, such a PC also receives feedforward MLI inhibition. f) Examples of Purkinje cell activity recorded, whose complex spike pattern suggests they also received an airpuff-related climbing fiber signal. g,h) A Purkinje cell disinhibited by a suppressing Purkinje cell in a feedforward motif. i) Example of a Purkinje cell that shows no specific CF-tuning but exhibits facilitatory simple spike modulation.

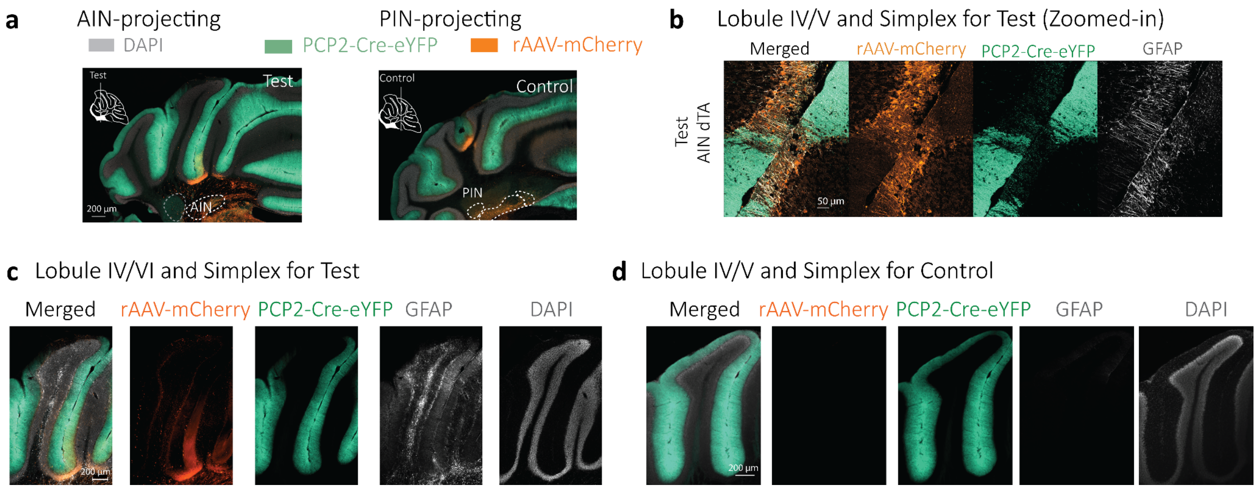

<u>Supplementary figure 7: Viral vector-assisted chronic lesion of Purkinje cells.</u> a) Injection site in the anterior interposed nucleus (AIN) for retrograde targeting of Purkinje cells in lobule simplex in mice expressing Cre in Purkinje cells (L7). rAAV-flex-dTA-mCherry (orange) construct was injected in AIN, leading to retrograde uptake and destruction of Purkinje cells (missing green) in lobule simplex following the Cre-dependent expression of diphtheria toxin A. Results shown here for one of four mice. b) A confocal image of the Simplex lobule for the test group shows that the presence of mCherry (orange), indicated viral particles, coincides with the absence of Purkinje cells (green), and the presence of a cell death marker glial fibrillary acidic protein (GFAP in white). c) Images of lobule IV/V and Simplex showing presence of viral particles (orange), signs of cell-death using the GFAP marker (white), Purkinje cells (green) and all nuclei marked in the cerebellar cortex with DAPI (gray). d) Same as c) but for the control group injected in the PIN.

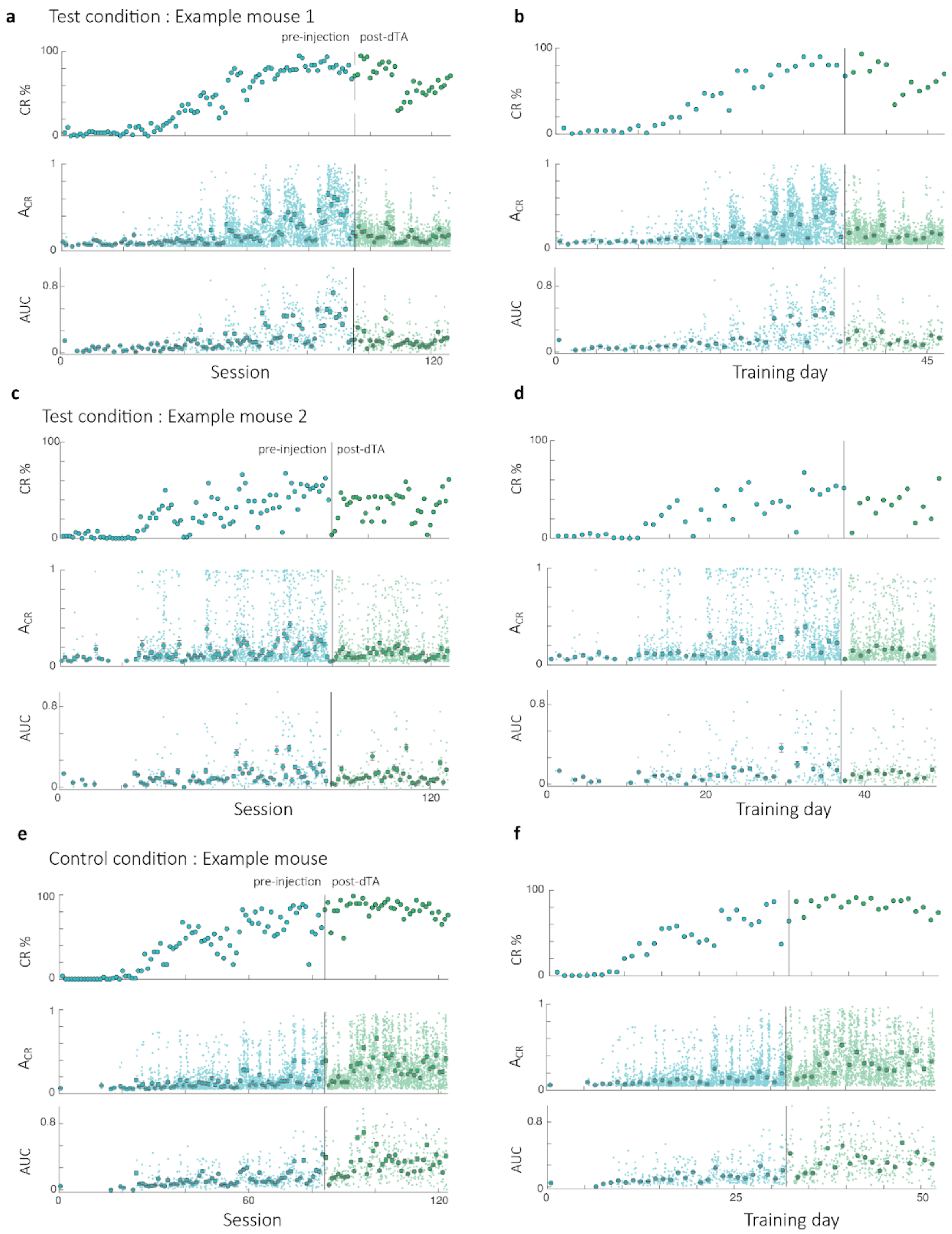

<u>Supplementary figure 8:</u> *a) Progression of various metrics across sessions for a uniform expert before (blue) and after (green) it received a viral construct injection carrying dTA in AIN, the test condition for lobule simplex. b) Data for same mouse plotted over*

training days. c,d) Same as a) and b) for another test mouse. e,f) same as a) and b) but for a control mouse where the viral construct was injected in the PIN, the control condition. Error bars represent standard error of mean.
