# Supplementary material for: Heterogeneous encoding of temporal stimuli in the cerebellar cortex
Source: Nat Commun. 2023 Nov 21;14:7581. doi: 10.1038/s41467-023-43139-9 (PMC10663630; doi:10.1038/s41467-023-43139-9)
Supplement: Supplementary file 3 — Reporting Summary [file 41467_2023_43139_MOESM3_ESM.pdf]

nature portfolio

Corresponding author(s): Devika Narain

Last updated by author(s): Oct 9, 2023

Reporting Summary

Nature Portfolio wishes to improve the reproducibility of the work that we publish. This form provides structure for consistency and transparency in reporting. For further information on Nature Portfolio policies, see our Editorial Policies and the Editorial Policy Checklist.

Statistics

For all statistical analyses, confirm that the following items are present in the figure legend, table legend, main text, or Methods section.

n/a	Confirmed	
☐	☒	The exact sample size (*n*) for each experimental group/condition, given as a discrete number and unit of measurement
☐	☒	A statement on whether measurements were taken from distinct samples or whether the same sample was measured repeatedly
☐	☒	The statistical test(s) used AND whether they are one- or two-sided<br>*Only common tests should be described solely by name; describe more complex techniques in the Methods section.*
☒	☐	A description of all covariates tested
☐	☒	A description of any assumptions or corrections, such as tests of normality and adjustment for multiple comparisons
☐	☒	A full description of the statistical parameters including central tendency (e.g. means) or other basic estimates (e.g. regression coefficient) AND variation (e.g. standard deviation) or associated estimates of uncertainty (e.g. confidence intervals)
☐	☒	For null hypothesis testing, the test statistic (e.g. *F*, *t*, *r*) with confidence intervals, effect sizes, degrees of freedom and *P* value noted<br>*Give P values as exact values whenever suitable.*
☒	☐	For Bayesian analysis, information on the choice of priors and Markov chain Monte Carlo settings
☒	☐	For hierarchical and complex designs, identification of the appropriate level for tests and full reporting of outcomes
☒	☐	Estimates of effect sizes (e.g. Cohen's *d*, Pearson's *r*), indicating how they were calculated

Our web collection on statistics for biologists contains articles on many of the points above.

Software and code

Policy information about availability of computer code

Data collection	Data collection was used by custom-built code in Objective C and Matlab (2020a) and OpenEphys. The microscope data was obtained using Zen 2.6 Pro.
Data analysis	Custom built analysis code was used in Matlab (2022a). Kilosort v2 was used for analysis of the electrophysiological data and Allen CCF for the histology.

For manuscripts utilizing custom algorithms or software that are central to the research but not yet described in published literature, software must be made available to editors and reviewers. We strongly encourage code deposition in a community repository (e.g. GitHub). See the Nature Portfolio guidelines for submitting code & software for further information.

Data

Policy information about availability of data

All manuscripts must include a data availability statement. This statement should provide the following information, where applicable:

- Accession codes, unique identifiers, or web links for publicly available datasets
- A description of any restrictions on data availability
- For clinical datasets or third party data, please ensure that the statement adheres to our policy

The data generated in this study have been deposited in the Dryad database under accession code xxxx (currently unknown)

Research involving human participants, their data, or biological material

Policy information about studies with human participants or human data. See also policy information about sex, gender (identity/presentation), and sexual orientation and race, ethnicity and racism.

Reporting on sex and gender	N/A
Reporting on race, ethnicity, or other socially relevant groupings	N/A
Population characteristics	N/A
Recruitment	N/A
Ethics oversight	N/A

Note that full information on the approval of the study protocol must also be provided in the manuscript.

Field-specific reporting

Please select the one below that is the best fit for your research. If you are not sure, read the appropriate sections before making your selection.

☒ Life sciences ☐ Behavioural & social sciences ☐ Ecological, evolutionary & environmental sciences

For a reference copy of the document with all sections, see nature.com/documents/nr-reporting-summary-flat.pdf

Life sciences study design

All studies must disclose on these points even when the disclosure is negative.

Sample size	Sample sizes were based on estimates from prior work within our lab (Ten Brinke et al 2015).
Data exclusions	Data from the mice were only excluded if mice were unable to perform both prior distribution conditions or if due to surgical/implant failure we were unable to continue experiments.
Replication	Experimental results were repeated and replicated for within-mice groups (N=6).
Randomization	Mice (litter-mates) were randomly allocated to the two experimental conditions (test or control) for the dTA experiment.
Blinding	Blinding was challenging to achieve in experiment 1 since the experimental condition becomes evident during the execution of the stimuli. Experimenters were blind to the group in the dTA experiment during the behavioral training.

Reporting for specific materials, systems and methods

We require information from authors about some types of materials, experimental systems and methods used in many studies. Here, indicate whether each material, system or method listed is relevant to your study. If you are not sure if a list item applies to your research, read the appropriate section before selecting a response.

Materials & experimental systems

n/a	Involved in the study	
☐	☒	Antibodies
☒	☐	Eukaryotic cell lines
☒	☐	Palaeontology and archaeology
☐	☒	Animals and other organisms
☒	☐	Clinical data
☒	☐	Dual use research of concern
☒	☐	Plants

Methods

n/a	Involved in the study	
☒	☐	ChIP-seq
☒	☐	Flow cytometry
☒	☐	MRI-based neuroimaging

Antibodies

Antibodies used	rabbit ant-GFAP, Agilent Technologies Inc., cat. no. GA52461-2; donkey-anti-rabbit Cy5, Jackson, cat. no. 711-175-152

Validation	Eng LF, Ghirnikar RS, Lee YL. Glial fibrillary acidic protein: GFAP-thirty-one years (1969-2000). Neurochem Res. 2000 Oct;25(9-10):1439-51.

Animals and other research organisms

Policy information about studies involving animals; ARRIVE guidelines recommended for reporting animal research, and Sex and Gender in Research

Laboratory animals	Both C57BL/6 mice as well as Bac2-Pcp2-IRES-Cre, Rosa26-LSL-ChR2-eYFP (C57BL/6 background) were used. n=14, 5 of which are female. All mice are used after postnatal day 60.
Wild animals	No wild animals are involved in this study.
Reporting on sex	Mice of both sex were used in all groups of this study.
Field-collected samples	No field-collected samples were used in this study.
Ethics oversight	Procedures are in accordance with protocols approved by animal care and use committees (IVD) at the Erasmus Medical Center.

Note that full information on the approval of the study protocol must also be provided in the manuscript.
